# Wear patterns and dental functioning in an Early Cretaceous stegosaur from Yakutia, Eastern Russia

**DOI:** 10.1371/journal.pone.0248163

**Published:** 2021-03-17

**Authors:** Pavel P. Skutschas, Vera A. Gvozdkova, Alexander O. Averianov, Alexey V. Lopatin, Thomas Martin, Rico Schellhorn, Petr N. Kolosov, Valentina D. Markova, Veniamin V. Kolchanov, Dmitry V. Grigoriev, Ivan T. Kuzmin, Dmitry D. Vitenko

**Affiliations:** 1 Vertebrate Zoology Department, Saint Petersburg State University, Universitetskaya Emb. 7/9, St. Petersburg, Russia; 2 Zoological Institute of the Russian Academy of Sciences, Universitetskaya Emb. 1, St. Petersburg, Russia; 3 Borissiak Paleontological Institute of the Russian Academy of Sciences, Profsouznaya ul. 123, Moscow, Russia; 4 Institute of Geosciences, Section Paleontology, Rheinische Friedrich-Wilhelms-Universität Bonn, Bonn, Germany; 5 Institute of Diamond and Precious Metals Geology, Siberian Branch of the Russian Academy of Sciences, Yakutsk, Russia; Universidad de Magallanes, CHILE

## Abstract

Isolated stegosaurian teeth from the Early Cretaceous high-latitude (palaeolatitude estimate of N 62°- 66.5°) Teete locality in Yakutia (Eastern Siberia, Russia) are characterized by a labiolingually compressed, slightly asymmetrical and mesiodistally denticulated (9–14 denticles) crown, a pronounced ring-like cingulum, as well as a “complex network of secondary ridges”. The 63 teeth (found during on-site excavation in 2012, 2017–2019 and screen-washing in 2017–2019) most likely belong to one species of a derived (stegosaurine) stegosaur. Most of the teeth exhibit a high degree of wear and up to three wear facets has been observed on a single tooth. The prevalence of worn teeth with up to three wear facets and the presence of different types of facets (including steeply inclined and groove-like) indicate the tooth-tooth contact and precise dental occlusion in the Teete stegosaur. The microwear pattern (mesiodistally or slightly obliquely oriented scratches; differently oriented straight and curved scratches on some wear facets) suggest a complex jaw mechanism with palinal jaw motion. Histological analysis revealed that the Teete stegosaur is characterized by relatively short tooth formation time (95 days) and the presence of a “wavy enamel pattern”. Discoveries of a “wavy enamel pattern” in the Teete stegosaur, in a Middle Jurassic stegosaur from Western Siberia, and in the basal ceratopsian *Psittacosaurus*, suggest that this histological feature is common for different ornithischian clades, including ornithopods, marginocephalians, and thyreophorans. A juvenile tooth in the Teete sample indicates that stegosaurs were year-round residents and reproduced in high latitudes. The combination of high degree of tooth wear with formation of multiple wear facets, complex jaw motions, relatively short tooth formation time and possibly high tooth replacement rates is interpreted as a special adaptation for a life in high-latitude conditions or, alternatively, as a common stegosaurian adaptation making stegosaurs a successful group of herbivorous dinosaurs in the Middle Jurassic–Early Cretaceous and enabeling them to live in both low- and high-latitude ecosystems.

## Introduction

The findings of polar dinosaurs have changed our understanding of dinosaurs as warm climate animals, but also have raised a number of questions. Were these dinosaurs perennial polar residents or did they migrate south with the beginning of the polar night? If they stayed during the polar winter, how could they survive it? Did the polar dinosaurs have any special adaptations not present in their relatives from lower latitudes? Most of these (and many other) questions have not yet been answered unequivocally, although there is significant progress in the study of polar dinosaurs and polar Mesozoic ecosystems (e.g., [1–8]).

The Early Cretaceous (Berriasian–Barremian) Teete vertebrate assemblage in southwestern Yakutia (Eastern Siberia, Russia) was formed close to Mesozoic polar latitudes (paleolatitude estimate of N 62° in Averianov et al., 2019 and N 66.5° in Fossilworks, http://fossilworks.org.) and is widely known for its polar dinosaurs, as well as turtles, salamanders, and mammaliaforms and mammals [7–14]. The high-latitude dinosaurian assemblage of Teete includes stegosaurs, ornithopods, theropods, and sauropods [9,10]. Stegosaurian remains are the most abundant and are represented by numerous isolated teeth, vertebrae, ribs, rare long bones, elements of shoulder and pelvic girdles, and few fragmentary cranial bones. Stegosaurian remains (namely teeth) from the Teete locality were first described and attributed to *Stegosaurus* sp. by Kurzanov et al., 2003 [15] who also noted that “the majority of teeth are strongly worn”. The identification of the Teete teeth by Kurzanov et al., 2003 [15] was preliminary and has been accepted by some subsequent workers, e.g. [16]. However, Maidment et al., 2008 [17] did not agree with the assignment of the Teete teeth to stegosaurs. They noted that “the large denticles that are confluent with striations extending from the base of the crown to the tip are unlike those seen on other stegosaurian dinosaurs” and referred them to an indeterminate ankylosaur [17]. A preliminary study of newly collected material has shown that the supposed thyreophoran teeth from Teete exhibit stegosaurian dental features (for details see “Affinities of Teete stegosaur” below) and Averianov et al., 2018 [9] identified a number of isolated teeth from Teete as Stegosauria indet.

Stegosaurs from the Teete locality are of particular interest for the following reasons: (1) they are among rare Early Cretaceous stegosaurs (other Early Cretaceous stegosaurs are known from Europe, South Africa and Asia; e.g. [17–19]); (2) they are the northernmost known stegosaurian record, and (3) represent a potentially important source of information on the paleobiology of polar dinosaurs.

The aims of this paper are to describe the morphology, macro- and microwear, and microanatomy of the stegosaur teeth from Teete, to reconstruct their feeding adaptation and to discuss paleobiological aspects of these high-latitude dinosaurs.

## Materials and methods

The studied stegosaur teeth were collected from the Batylykh Formation, Sangar Series (Lower Cretaceous, Berriasian–Barremian) at the Teete locality in Suntar Ulus, Yakutia, Eastern Siberia, Russia. For the geological settings and detailed description of the Teete section see Averianov et al., 2018 [9]. On-site excavation was employed in 2012 and 2017–2019. Additionally, we screen-washed fossiliferous matrix (500 kg of matrix in 2017, 675 kg in 2018, and 150 kg in 2019). A total of 63 isolated stegosaurian teeth from the Teete locality were examined, comprising almost all available teeth with preserved crowns from this locality. Among the studied teeth, only six have a fully unworn crown or exhibit an initial stage of wear expressed by a slight abrasion of the apex of the median denticle, while 57 teeth (90.5%) bear one or more distinct and pronounced wear facets. The material is housed in the Paleoherpetological Collection of the Zoological Institute of the Russian Academy of Sciences, Saint Petersburg, Russia (ZIN PH).

**Fig 1:**
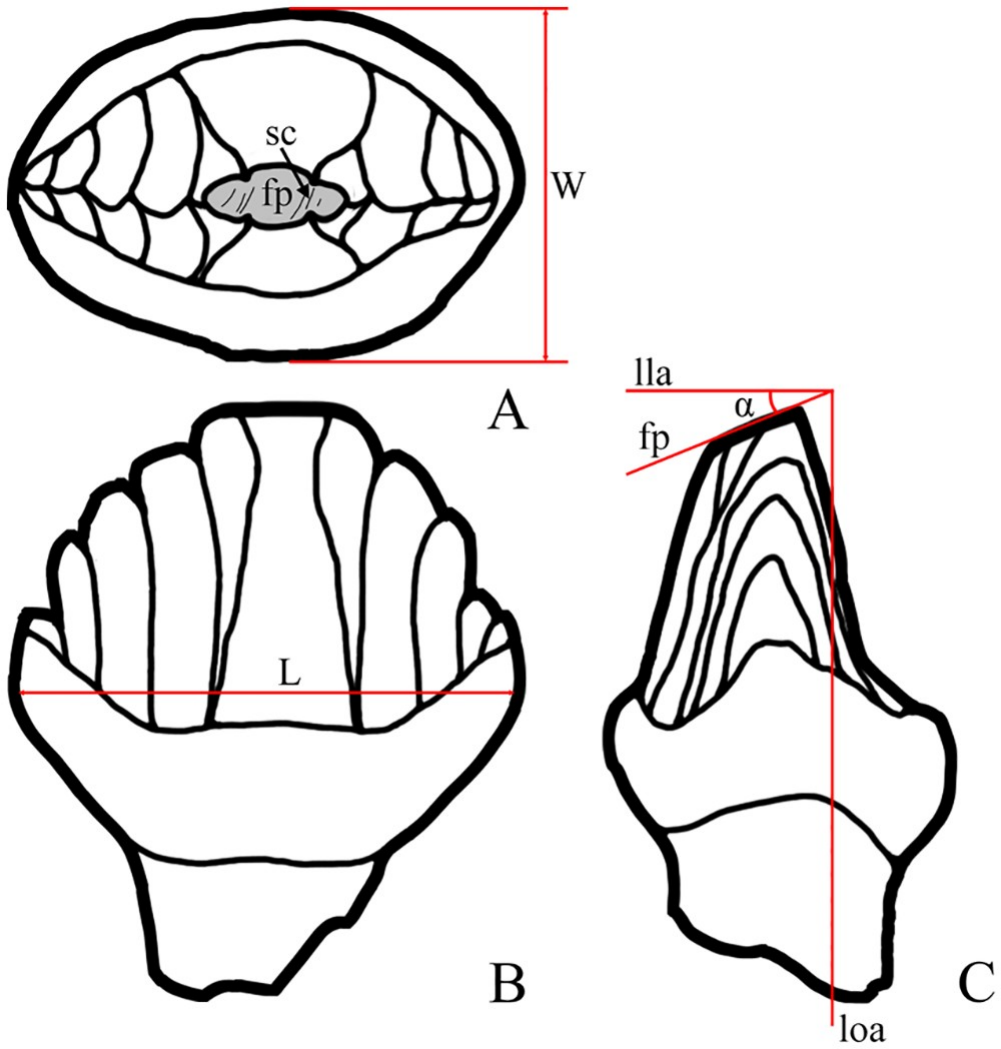
Diagrams illustrating measurements and wear elements of isolated stegosaur teeth. Stegosaur tooth in occlusal view (A), labial or lingual view (B), and mesial or distal view (C). Abbreviations: W, crown width; L, crown length; fp, facet plane; sc, wear scratches; lla, labiolingual axis of the crown; loa, longitudinal axis of the crown; α, facet angle.

The wear facet angle (*α*) was measured between the wear facet plane and the labiolingual tooth axis, which is perpendicular to the longitudinal tooth axis (Fig 1). The angle *α* was measured by protractor to the nearest 1°. Additionally, we measured the mesiodistal length (L) and labiolingual width (W) of the crown (Fig 1; for measurements of studied teeth see S1 and S2 Files). All measurements are in millimeters.

Four specimens (ZIN PH 63-66/246) with unworn or slightly worn crowns were μCT scanned (at 100 kV and 0.1 mA, generating a resolution of 9 μm pixel size and an output of 2960x2960 pixels per slice) at the Saint Petersburg State University Research Centre for X-ray Diffraction Studies (Saint Petersburg, Russia) using a Skyscan 1172. Segmentation of the μCT scan data and 3D model reconstructions were made with Amira 6.3.0 (FEIVSG Company).

For the description of the histology, we sampled five fragmentary tooth crowns, but thin sections of only one specimen (relatively large and presumably adult specimen ZIN PH 41/246) allowed to document the Lines of von Ebner correlating to tooth age in dinosaurs (see Erickson, 1996 [20]). Additionally, for histological comparisons, we sampled the tooth ZIN PH 104/117 of Stegosauria indet. from the Middle Jurassic Itat Formation (Berezovsk coal mine, Krasnoyarsk Territory, Western Siberia, Russia, see [21]) and an unnumbered tooth of the primitive ceratopsian *Psittacosaurus* from the Lower Cretaceous Ilek Formation (Shestakovo locality, Kemerovo Province, Western Siberia, Russia, see [22]). The sections were examined under normal and polarized light using an optical microscope (Leica 4500, Leica Microsystems, Wetzlar, Germany) in the Saint Petersburg State University Research Centre for X-ray Diffraction Studies (Saint Petersburg, Russia). The thin-sections used in this study are housed in the histological collection of the Department of Vertebrate Zoology, Saint Petersburg State University, Saint Petersburg, Russia (DVZ).

## Results

### Identification of teeth

**Fig 2:**
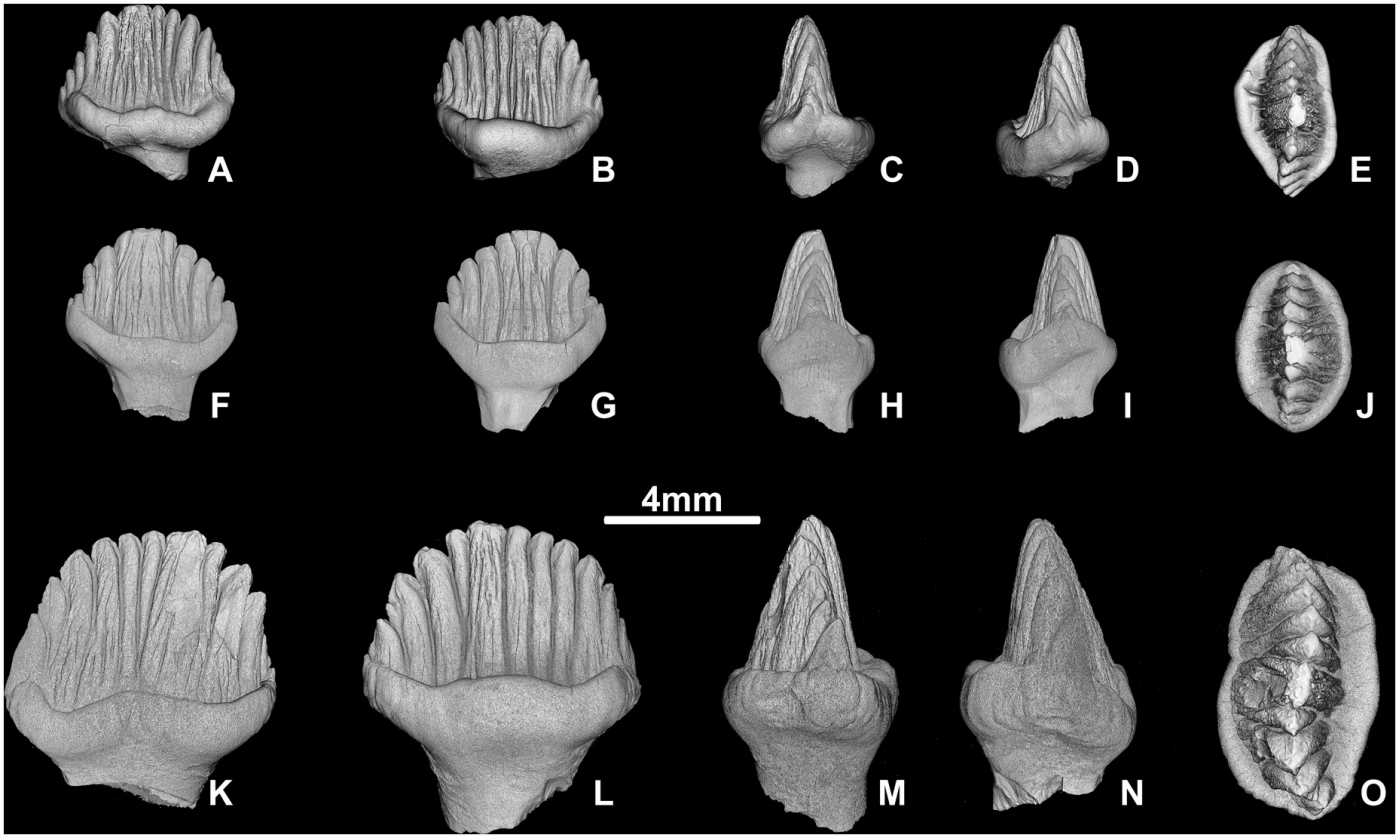
Unworn teeth of Stegosauria indet. from the Teete locality, Yakutia, Russia; Batylykh Formation (Lower Cretaceous). Digital restoration of maxillary tooth ZIN PH 63/246 (A-E) and dental teeth ZIN PH 65/246 (F-J) and ZIN PH 64/246 (K-O) in lingual (A, F, K), labial (B, G, L), distal? (C, H, N), mesial? (D, I, M) and occlusal (E, J, O) views.

The cheek teeth of stegosaurs have a similar morphology but a slight asymmetry allows to distinguish the mesial and distal ends as well as maxillary from dentary teeth at least in *Stegosaurus* [18,23–25]. The mesial part of the crown has fewer and larger marginal denticles and a convex outline in labial or lingual view. Subsequently, the distal part of the crown has smaller marginal denticles and a concave outline in labial or lingual view. The cingulum is usually more pronounced on the labial side of both maxillary and dentary teeth. The crowns of maxillary teeth curve slightly lingually, while the crowns of dentary teeth curve labially. This allows to distinguish unworn or partly worn maxillary and dentary teeth (see Fig 2). As in other thyreophorans [26,27], in stegosaurs the maxillary tooth row is situated labially to the dentary tooth row in occlusion and, respectively, the wear facet is on the lingual crown side in maxillary teeth and on the labial crown side in dentary teeth. There are some teeth in the sample with a cingulum equally developed on both crown sides. Attribution of these teeth to the upper or lower jaw is not possible.

### General morphology of teeth

The tooth crowns are covered with enamel on both sides. The crowns of the cheek teeth are about 1.5 times mesiodistally longer than labiolingually wide. The mesiodistal length (L) of the maxillary teeth varies from 4.4 mm to 7.2 mm (*M* = 5.7) and the labiolingual width (W) from 2.9 mm to 5.3 mm (*M* = 3.9). The mesiodistal length (L) of the dentary teeth varies from 4.3 mm to 8.3 mm (*M* = 6.4) and the labiolingual width (W) from 2.4 mm to 5.3 mm (*M* = 3.8).

The crown bears marginal denticles and associated ridges that extend down to the crown base and to the ring-like cingulum all around the crown base (Fig 2). The median or primary ridge, bearing the highest denticle, is usually more pronounced than the side ridges on the lingual side, while on the labial side it can be similar in width with the adjacent side denticles. In most teeth the crown is slightly asymmetrical, with three-five larger denticles on the mesial side and four-seven smaller denticles on the distal side. However, there are more symmetrical crowns, with four denticles on each side (dentary teeth ZIN PH 27 and 33/246). The crown is also asymmetrical in the labiolingual plane: one side is slightly convex while the opposite side is flat or slightly concave. The convex side is labial in the maxillary teeth and lingual in the dentary teeth. In occlusal view, the labial crown side, which bears a thicker cingulum, is more or less convex on all maxillary and dentary teeth. In 13 maxillary and 12 dentary teeth the lingual crown side is also convex. In 13 maxillary and seven dentary teeth there is a variously developed concavity on the lingual crown side. The lingual crown side can be almost straight (one maxillary and nine dentary teeth) or sinusoidal (two dentary teeth).

The marginal denticles are coarse and rounded. The smallest side denticles are associated with the cingulum (Fig 2). The ridges are covered by a complex network of secondary ridges that was reported previously only for *Stegosaurus* [18,24] and for an unnamed formally stegosaur taxon from the Middle Jurassic Berezovsk Quarry locality in Western Siberia [28]. Usually there are two or three fine ridges associated with the marginal denticles. On the median ridge, the number of secondary fine ridges is larger and varies depending on the width of the primary ridge. The secondary ridges are more numerous on the lingual side, where the median ridge is wider. In one maxillary and six dentary teeth the primary ridge is offset from the row of other ridges and extends more lingually; on the labial side, there is a groove associated with the primary ridge. The cingulum consists of a central horizontal part and side wards ascending parts. On the lingual crown side of 17 maxillary and ten dentary teeth, the horizontal part of the cingulum is upwardly arched to various extent, with convex apical side and concave basal side. The cingulum is straight in ten maxillary and 18 dentary teeth. On the labial crown side, the horizontal part of the cingulum is straight in most specimens. It is upwardly arched in six maxillary and six dentary teeth. On the labial side, the cingulum is thicker and usually separated from the rest of the crown by a deep groove. In one dentary and three maxillary teeth the labial cingulum is disproportionally large. On the lingual crown side, the groove separating the cingulum from the cusps is much shallower along the ascending parts of the cingulum and absent in the middle, opposite the median ridge. On some specimens the cingulum is interrupted on the lingual side at the median ridge. At the mesial and distal end the cingulum sometimes forms small denticles which are in line with the marginal denticles of the crown. The root is distinctly narrower mesiodistally than the crown. When the complete root is preserved, it comprises 70–80% of the total tooth height. The root tapers gradually towards the distal end. It is oval in cross section, constricted at the crown-root junction, and swollen at midheight.

### Tooth wear patterns

#### Maxillary teeth

**Fig 3:**
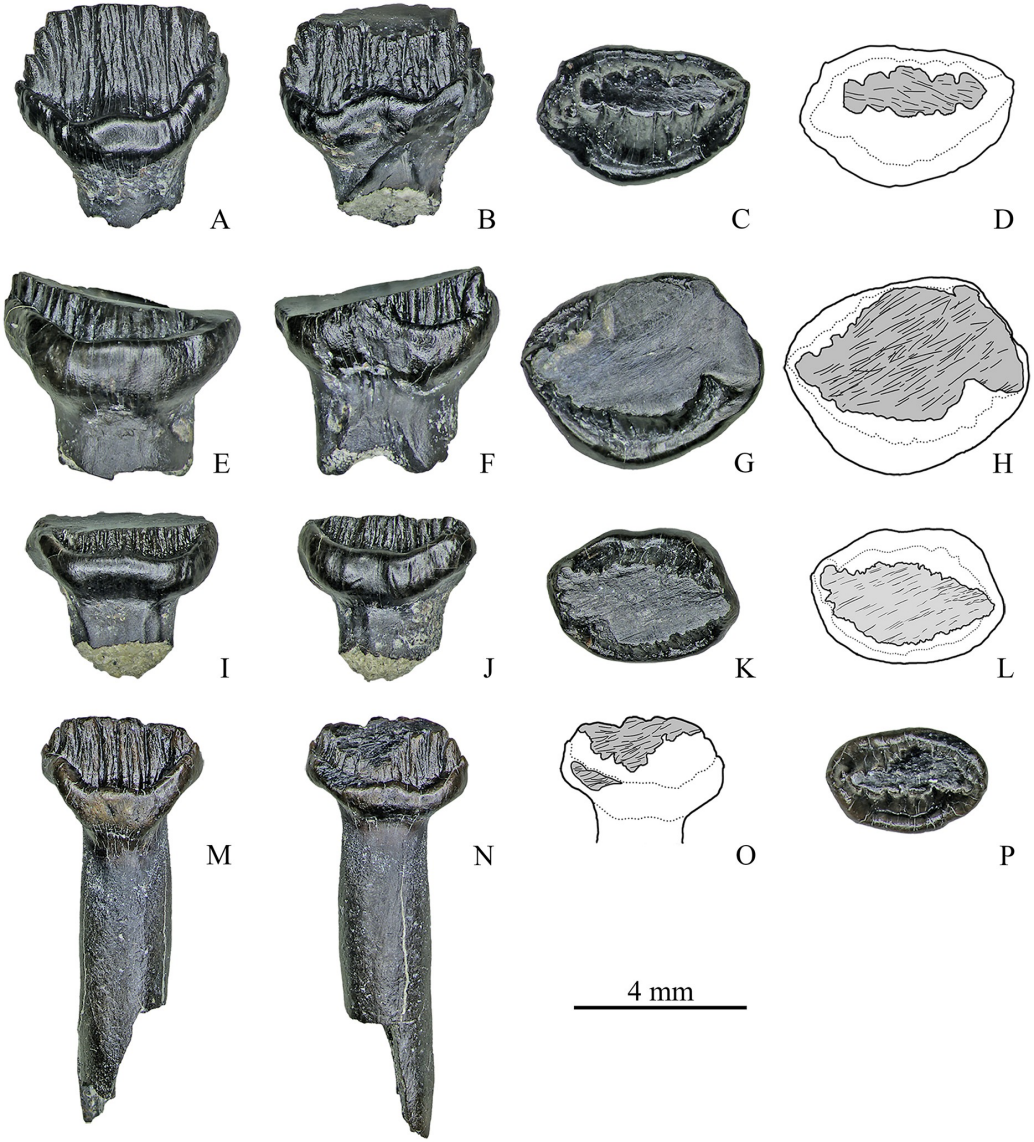
Maxillary teeth with a single apical facet of Stegosauria indet. from the Teete locality, Yakutia, Russia; Batylykh Formation (Lower Cretaceous). Specimens ZIN PH 7/246 (A–D), ZIN PH 11/246 (E–H), ZIN PH 20/246 (I–L), ZIN PH 6/246 (M–P) in labial (A, E, I, M), lingual (B, F, J, N), occlusal (C, G, K, P) views, and interpretive drawings in occlusal (D, H, L) and lingual (O) views showing the wear facets with scratches.

**Fig 4:**
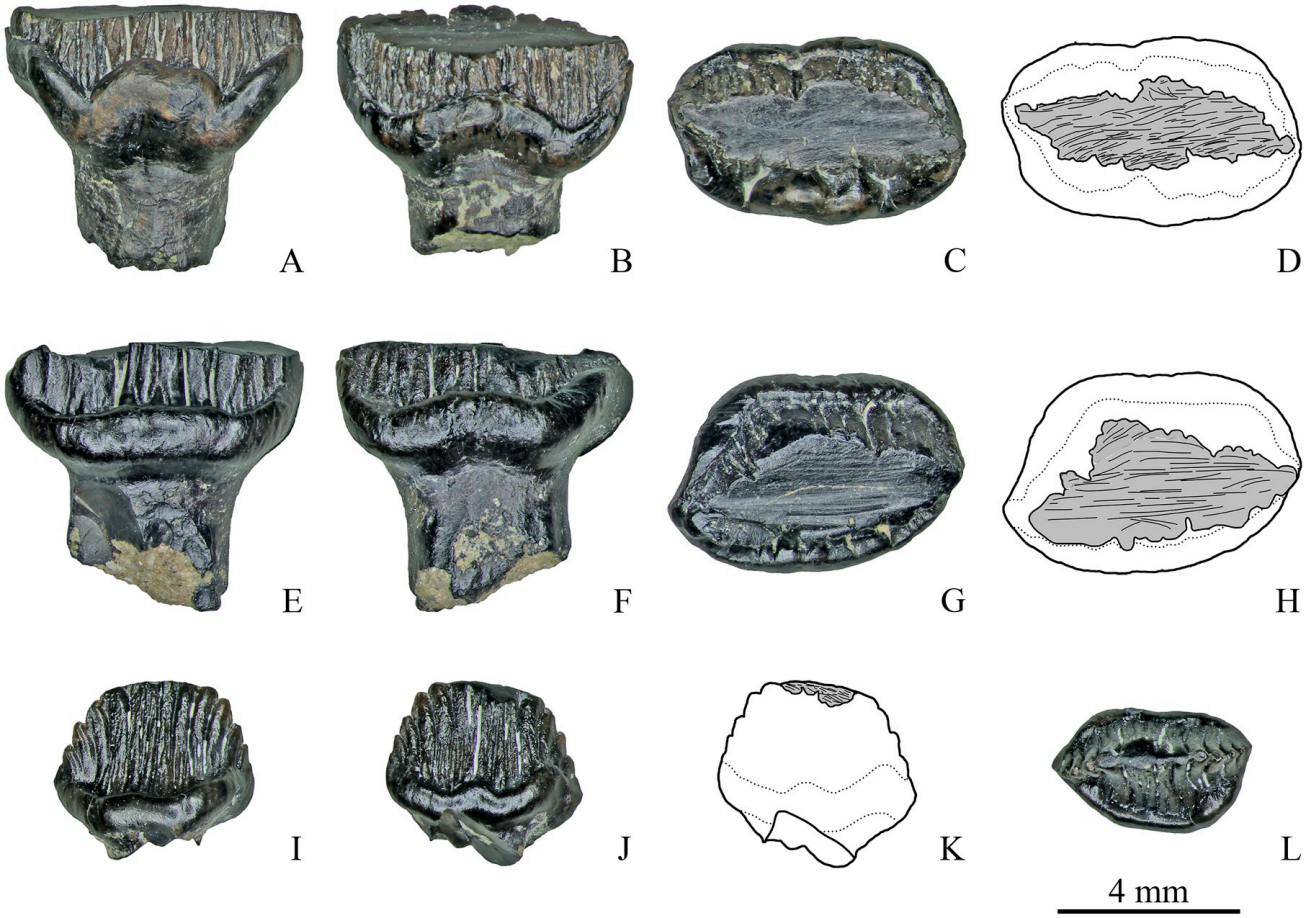
Maxillary teeth with a single apical facet of Stegosauria indet. from the Teete locality Yakutia, Russia; Batylykh Formation (Lower Cretaceous). Specimens ZIN PH 55/246 (A–D), ZIN PH 15/246 (E–H), ZIN PH 58/246 (I–L) in labial (A, E, I), lingual (B, F, J), occlusal (C, G, L) views, and interpretive drawings in occlusal (D, H) and lingual (K) views showing the wear facets with scratches.

In the sample of 27 worn maxillary teeth, the main type of wear facets is single apical (*n* = 18). The wear facets of this type are situated on the apical region of the crown or extend from the apex to the base of the crown (Figs 3 and 4). The wear facet angle (*α*) varies up to 41° (*M* = 20.5°). Size of wear facets depends on the degree of wear: wear facets occur only on the apical region of the crown on slightly worn teeth, while the crown apices are abraded and wear facets extend onto the cingulum on highly worn teeth. The facets bear parallel, straight, mesiodistally or obliquely (up to 30° to the mesiodistal axis) oriented scratches.

**Fig 5:**
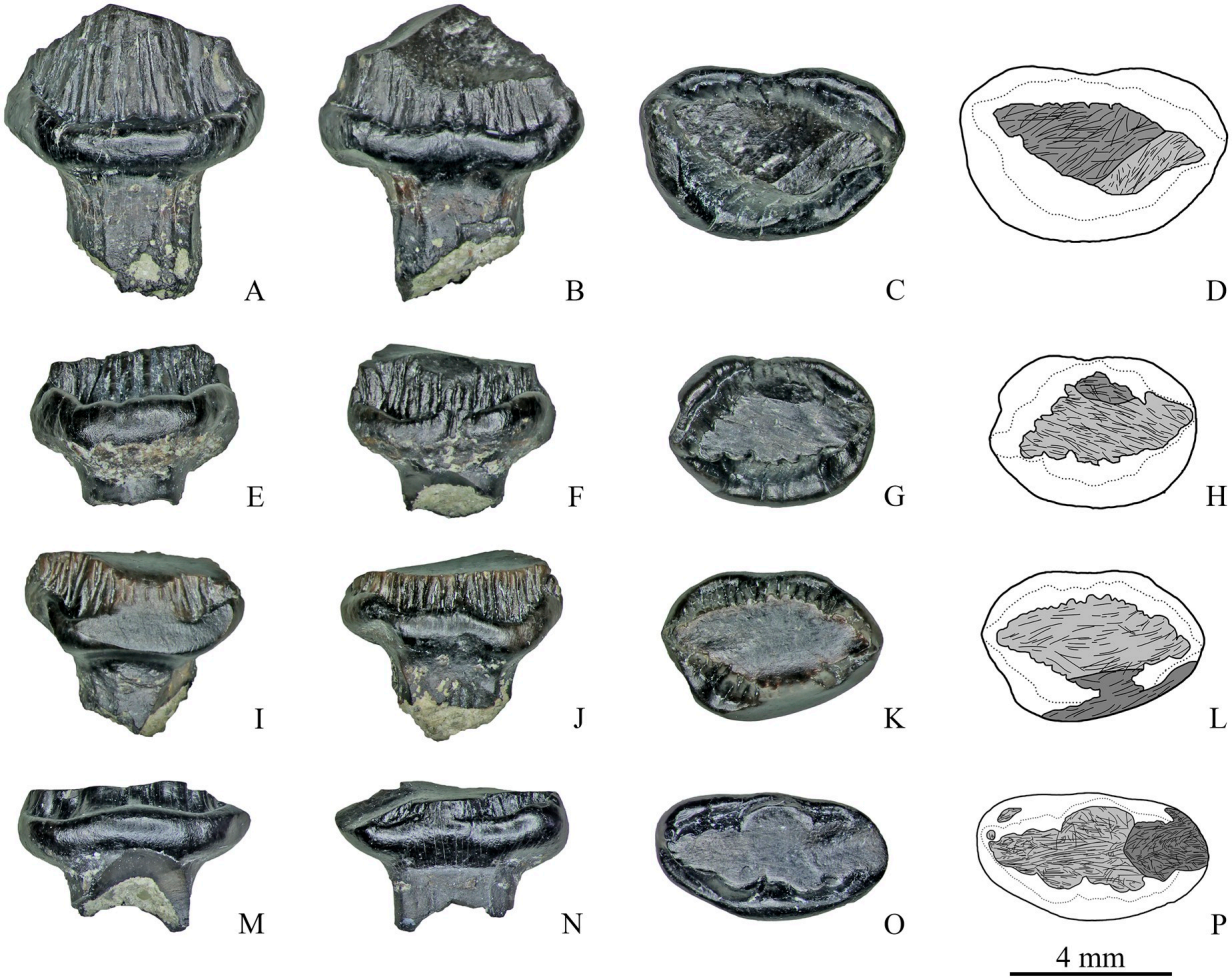
Maxillary teeth with two wear facets of Stegosauria indet. from the Teete locality, Yakutia, Russia; Batylykh Formation (Lower Cretaceous). Specimens ZIN PH 28/246 (A-D), ZIN PH 31/246 (E-H), ZIN PH 53/246 (I-L), ZIN PH 69/246 (M-P) in labial (A, E, I, M), lingual (B, F, J, N), occlusal (C, G, K, O) views, and interpretive drawings (D, H, L, P) in occlusal views showing the wear facets with scratches.

**Fig 6:**
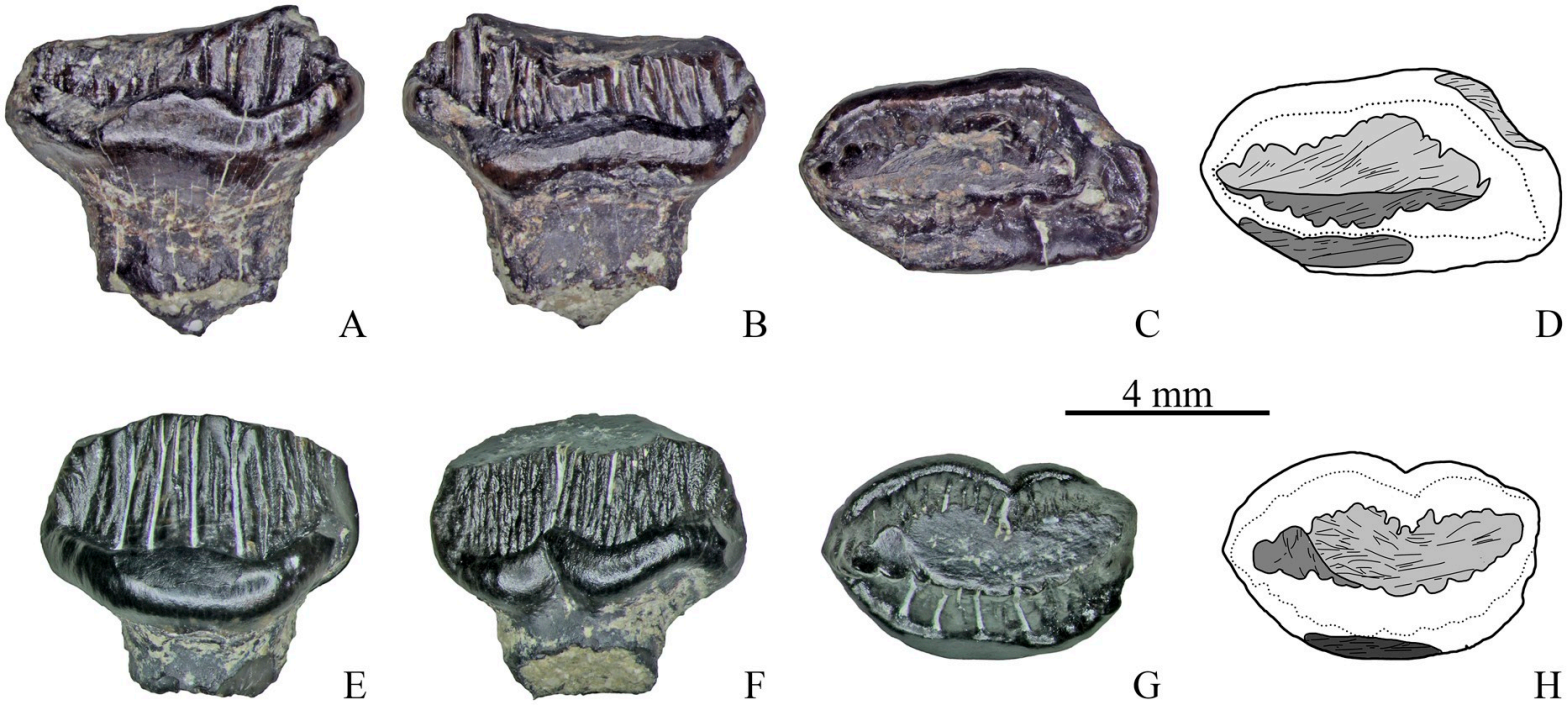
Maxillary teeth with two (A-D), and with three wear facets (E-H) of Stegosauria indet. from the Teete locality, Yakutia, Russia; Batylykh Formation (Lower Cretaceous). Specimens ZIN PH 80/246 (A-D), ZIN PH 57/246 (E-H) in labial (A, E), lingual (B, F), occlusal (C, G) views, and interpretive drawings (D, H) in occlusal views showing the wear facets with scratches.

Eight worn maxillary teeth exhibit two distinct apical wear facets (Figs 5 and 6A–6D). On seven teeth, one of these facets is horizontal or low-angled (*α* varies up to 18°; *M* = 12°) and the second is more steeply inclined (*α* varies from 29° to 64°; *M* = 39.25°) (Fig 5). The two facets come into contact with each other. Scratches on the facets are similar to those on the single apical wear facets. One specimen (ZIN PH 80/246, Fig 6A–6D) bears facets on both crown surfaces that are situated opposite to each other.

Specimen ZIN PH 57/246 exhibits three wear facets: two apical and one on the cingulum (Fig 6E–6H).

#### Dentary teeth

Among 28 worn dentary teeth, nine teeth bear a single apical wear facet, 17 worn dentary teeth exhibit two distinct apical wear facets and two specimens (ZIN PH 13/246, 19/246) have three facets.

**Fig 7:**
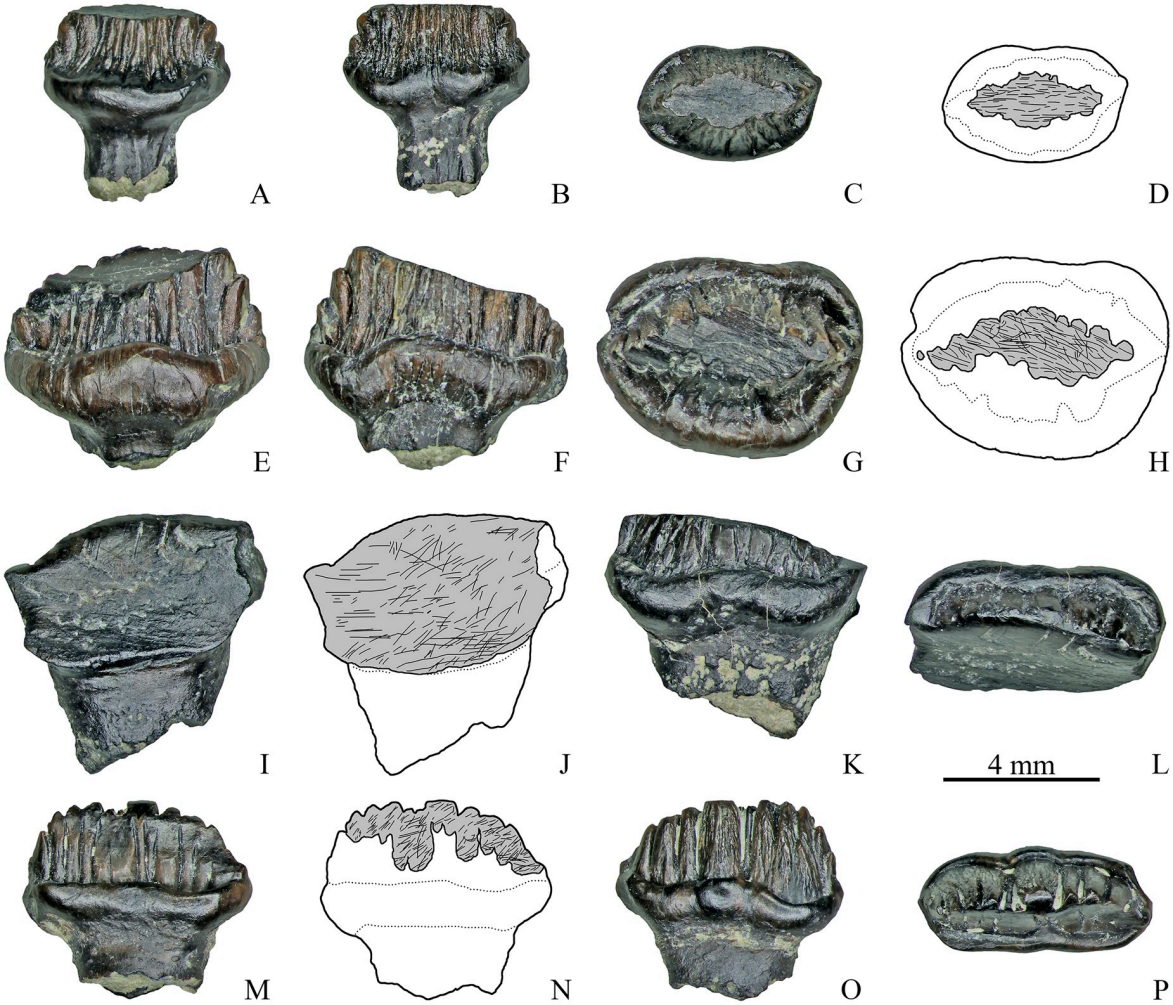
Dentary teeth with single apical wear facet of Stegosauria indet. from the Teete locality, Yakutia, Russia; Batylykh Formation (Lower Cretaceous). Specimens ZIN PH 23/246 (A–D), ZIN PH 54/246 (E–H), ZIN PH 26/246 (I–L), ZIN PH 56/246 (M-P) in labial (A, E, I, M), lingual (B, F, K, O), occlusal (C, G, L, P) views, and interpretive drawings in occlusal (D, H) and labial (J, N) views showing the wear facets with scratches.

The wear facet angle (*α*) in the teeth with a single facet varies from 9° to 69° (*M* = 35.8°) (Fig 7). The single facets bear parallel, straight, mesiodistally or obliquely (up to 50° to the mesiodistal axis) oriented scratches. One specimen (ZIN PH 26/246, Fig 7I–7L) with steeply inclined extensive facet on the strongly abraded crown bears straight, subparallel scratches in the more basal part of the facet (at the level of the abraded cingulum) and differently oriented (straight subvertical, straight oblique and curved) wide and deep grooves on the more apical part.

**Fig 8:**
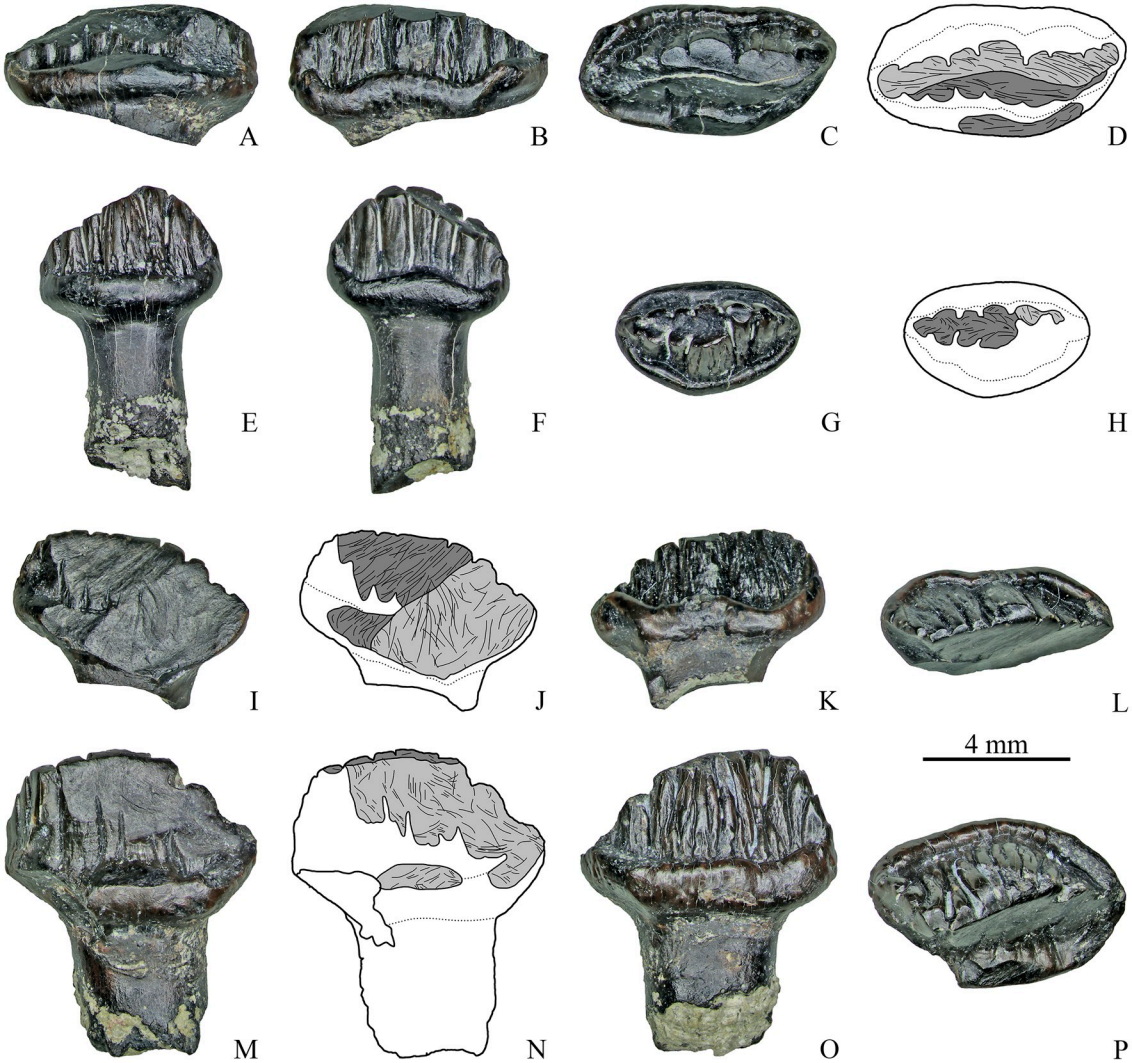
Dentary teeth with two wear facets of Stegosauria indet. from the Teete locality, Yakutia, Russia; Batylykh Formation (Lower Cretaceous). Specimens ZIN PH 16/246 (A-D), ZIN PH 27/246 (E-H), ZIN PH 22/246 (I-L), ZIN PH 50/246 (M-P) in labial (A, E, I, M), lingual? (B, F, K, O), occlusal (C, G, L, P) views, and interpretive drawings in occlusal (D, H) and labial (J, N) views showing the wear facets with scratches.

Among the teeth with two wear facets (*n* = 17), eight teeth have one low-angled (*α* varies up to 29°; *M* = 16.4°) and one more steeply inclined facet (*α* varies from 26° to 69°; *M* = 57.9°) (Fig 8). The shape and orientation of the scratches are similar to that of the single wear facets. One tooth (ZIN PH 22/246, Fig 8I–8L) has two extensive and steeply inclined facets (*α* is about 67°) that differ from each other in microwear pattern: one facet contains long, straight, parallel and obliquely oriented (at the angle about 50° to the vertical plane) scratches, while the other facet bears differently oriented scratches (oblique, subvertical and curved). The extensive steeply inclined facet seen in some specimens (ZIN PH 50/246, ZIN PH 52/246) has obliquely oriented parallel scratches in the region of the cingulum, but in the more apical part of this facet curved and subvertical scratches can occur (Fig 8M–8P).

**Fig 9:**
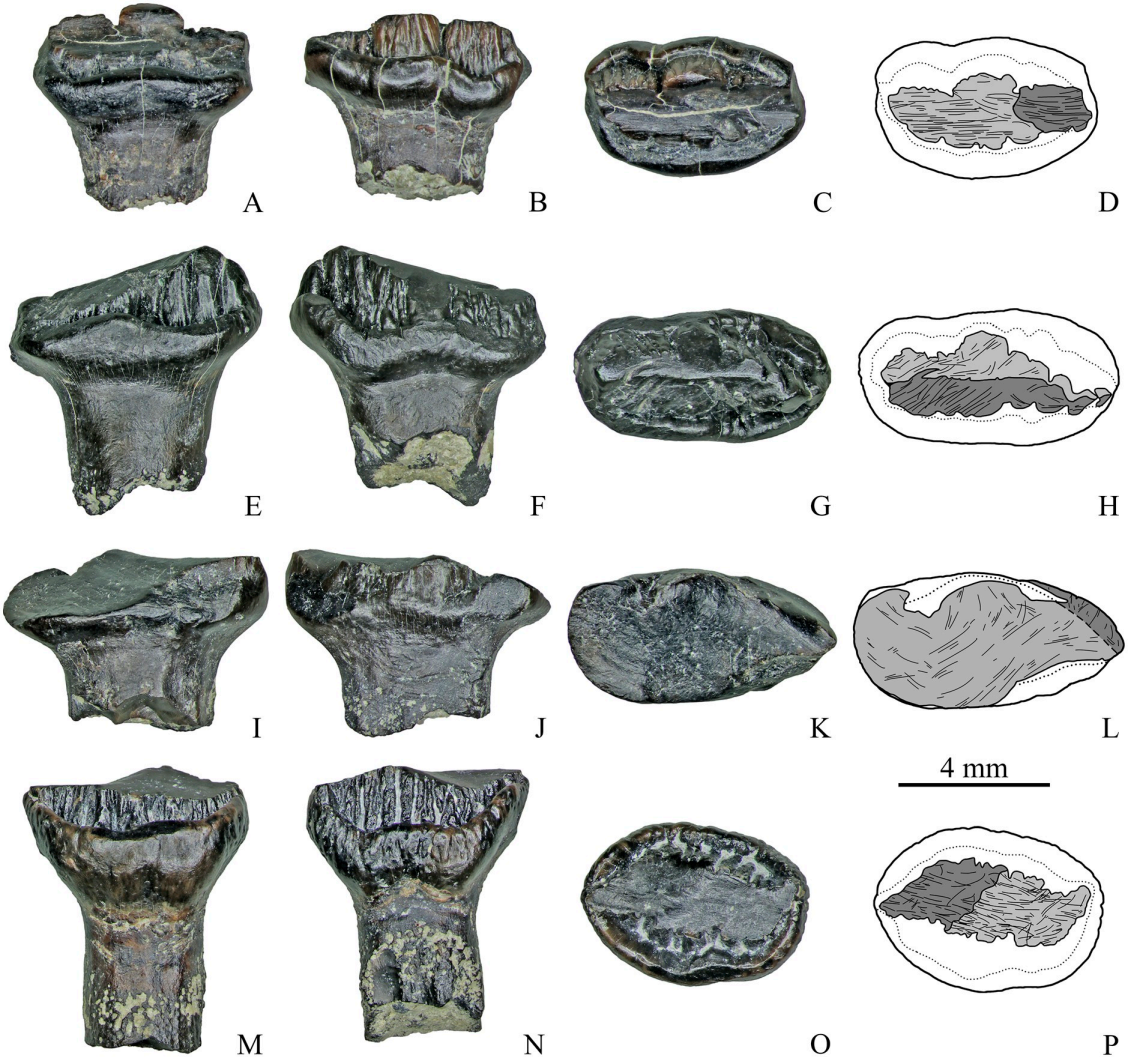
Dentary teeth with two wear facets of Stegosauria indet. from the Teete locality, Yakutia, Russia; Batylykh Formation (Lower Cretaceous). Specimens ZIN PH 12/246 (A-D), ZIN PH 21/246 (E-H), ZIN PH 25/246 (I-L), ZIN PH 49/246 (M-P) in labial (A, E, I, M), lingual (B, F, J, N), occlusal (C, G, K, O) views, and interpretive drawings in occlusal (D, H, L, P) view showing the wear facets with scratches.

Among the other teeth with two wear facets, three teeth have groove-like facets and five teeth have facets on both crown surfaces. The groove-like facets are either arranged mesiodistally with closely packed, straight, coarse, parallel and obliquely oriented scratches (up to about 30° the mesiodistal axis) (Fig 9A–9D) or the groove-like facet bearing similar scratches crosses an extensive and steeply inclined facet in its apical portion (ZIN PH 81/246).

The wear facets situated on both crown surfaces of several teeth (*n* = 5, Fig 9E–9P) vary in angle, size and form: in some teeth these facets have nearly the same size and are situated opposite to each other (ZIN PH 21/246, ZIN PH 72/246, Fig 9E–9H) or they are arranged mesiodistally (ZIN PH 49/246, Fig 9M–9P); in some specimens, they differ in size and shape (ZIN PH 25/246; ZIN PH 79/246, Fig 9I–9L). The shape and orientation of the scratches are similar to those of the other dentary teeth (closely packed, straight, coarse, parallel and obliquely oriented).

**Fig 10:**
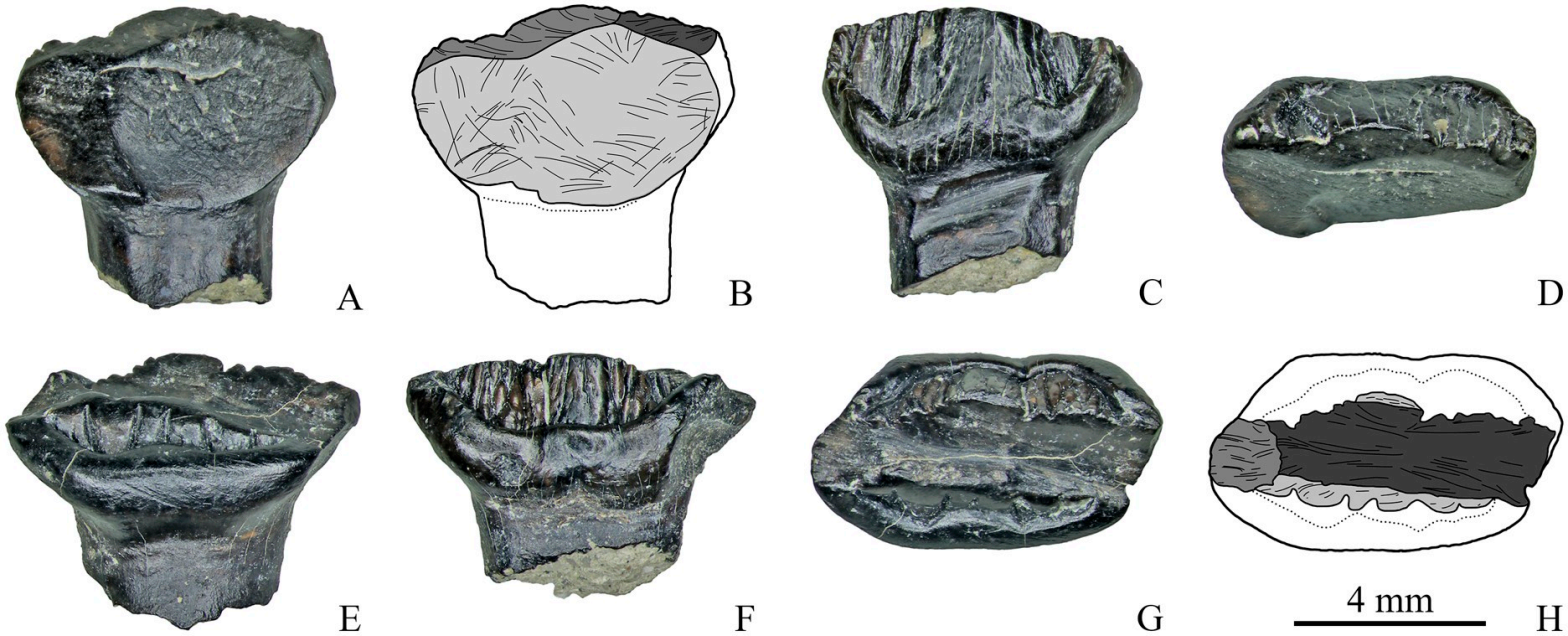
Dentary teeth with three wear facets of Stegosauria indet. from the Teete locality, Yakutia, Russia; Batylykh Formation (Lower Cretaceous). Specimens ZIN PH 13/246 (A-D), ZIN PH 19/246 (E-H) in labial (A, E), lingual (C, F), occlusal (D, G) views, and interpretive drawings in labial (B) and occlusal (H) views showing the wear facets with scratches.

Two specimens (ZIN PH 13/246 and 19/246) exhibit three wear facets. In specimen ZIN PH 13/246 (Fig 10A–10D) two facets are situated more apical and are relatively narrow, while one facet is extensive and reaches the level of the completely abraded cingulum. The basal portion of the extensive facet bears differently oriented straight and curved scratches.

Specimen ZIN PH 19/246 also has three wear facets: two mesiodistally arranged groove-like facets with closely packed, straight, coarse, parallel and obliquely oriented scratches (up to about 30° the mesiodistal axis) and one apical facet (Fig 10E–10H).

When two (or three) facets are present, they come into contact with each other or overlap.

#### Teeth of unknown position

**Fig 11:**
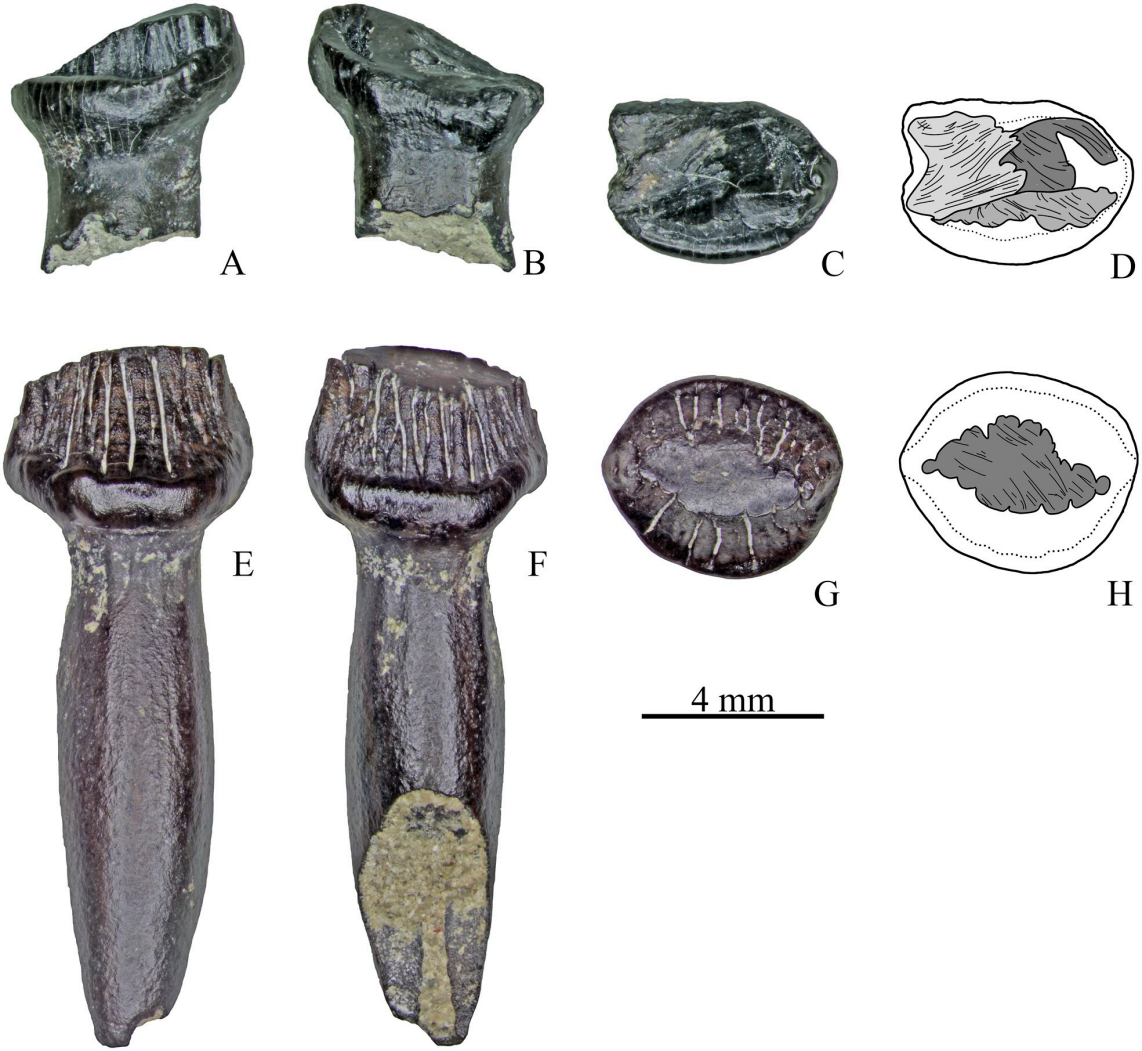
Teeth of Stegosauria indet. from the Teete locality Yakutia, Russia; Batylykh Formation (Lower Cretaceous) of unknown jaw positions. Specimens ZIN PH 14/246 (A-D), ZIN PH 71/246 (E-H) in labial or lingual (A-B; E, F), occlusal (C, G), and interpretive drawings in occlusal (D, H) views showing the wear facets with scratches.

Two teeth in the sample (Fig 11) cannot be attributed to the upper or lower jaw because of the strongly abraded crown (ZIN PH 14/246; Fig 11A–11D) or because the cingulum is equally developed on both crown sides (ZIN PH 71/246; Fig 11E–11H). ZIN PH 71/246 has a single apical wear facet (Fig 11E–11H). This facet bears parallel, straight, mesiodistally or obliquely (up to 45° to the mesiodistal axis) oriented scratches (Fig 11G and 11H).

The strongly worn tooth ZIN PH 14/246 (Fig 11A–11D) has two apical wear facets, one low-angled (*α* = 17°) and a second more steeply inclined (*α* = 26°), and two distinct small groove-like wear facets on the abraded area. The small groove-like wear facets bear closely packed, straight, coarse, parallel and obliquely oriented scratches (Fig 11C and 11D).

### Tooth microanatomy and histology

**Fig 12:**
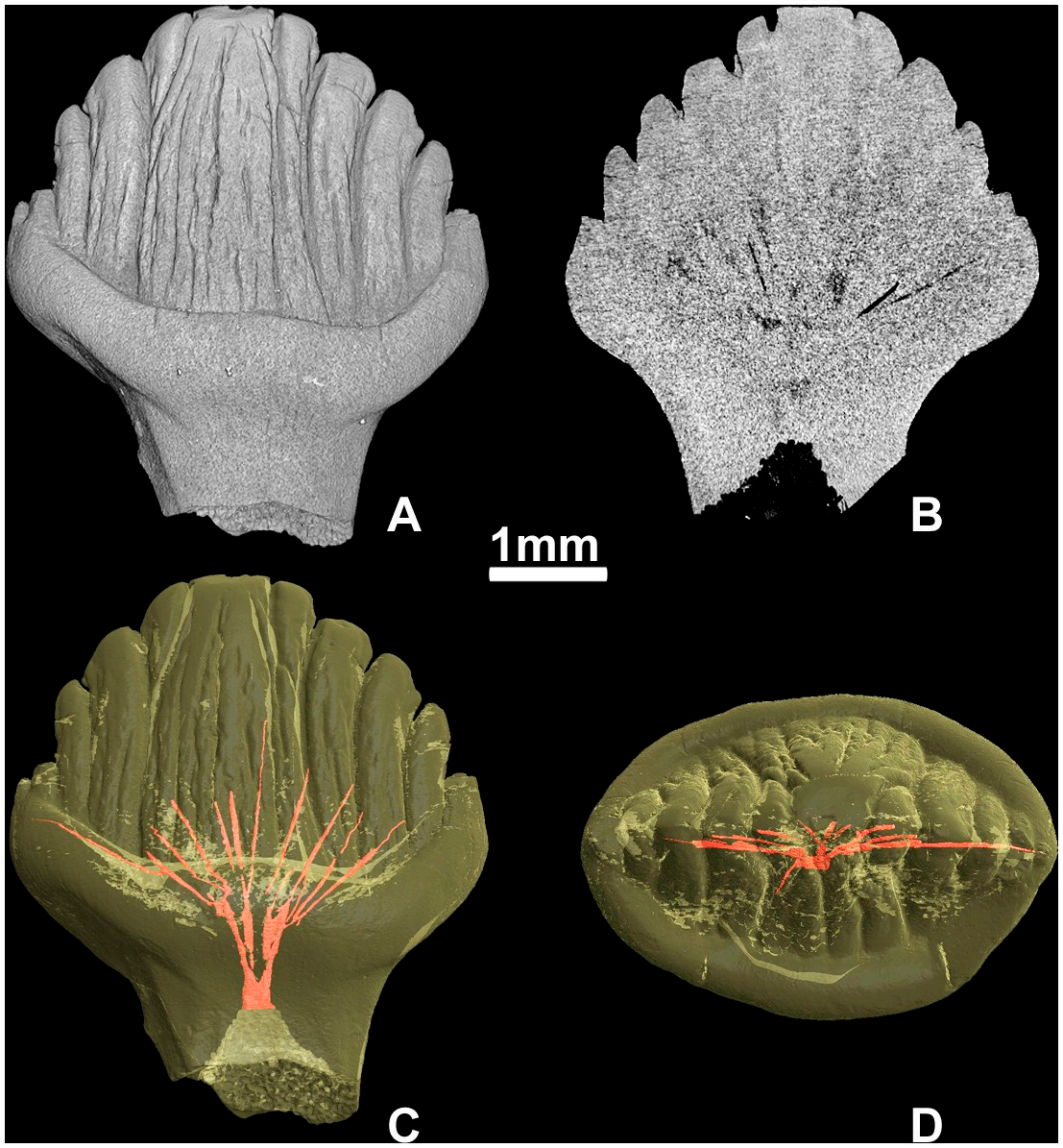
Tooth microanatomy of Stegosauria indet. from the Teete locality, Yakutia, Russia; Batylykh Formation (Lower Cretaceous). Digital restorations of the unworn tooth ZIN PH 65/246 in lingual view (A), vertical cross-section (B), in lingual view with visualized pulp cavity and diverging canals (C) and in occlusal view with visualized pulp and diverging canals (D).

The stegosaurian teeth from Teete have a well-defined pulp cavity at the base of the crown (Fig 12). In this region the pulp cavity is strongly labiolingually compressed. More apically, there are several canals diverging from the pulp cavity. Like the pulp cavity, these canals are situated along the mesiodistal plane of the crown. The pulp cavity and pulp canals are surrounded by dentin and a rather thin exterior cover of enamel.

**Fig 13:**
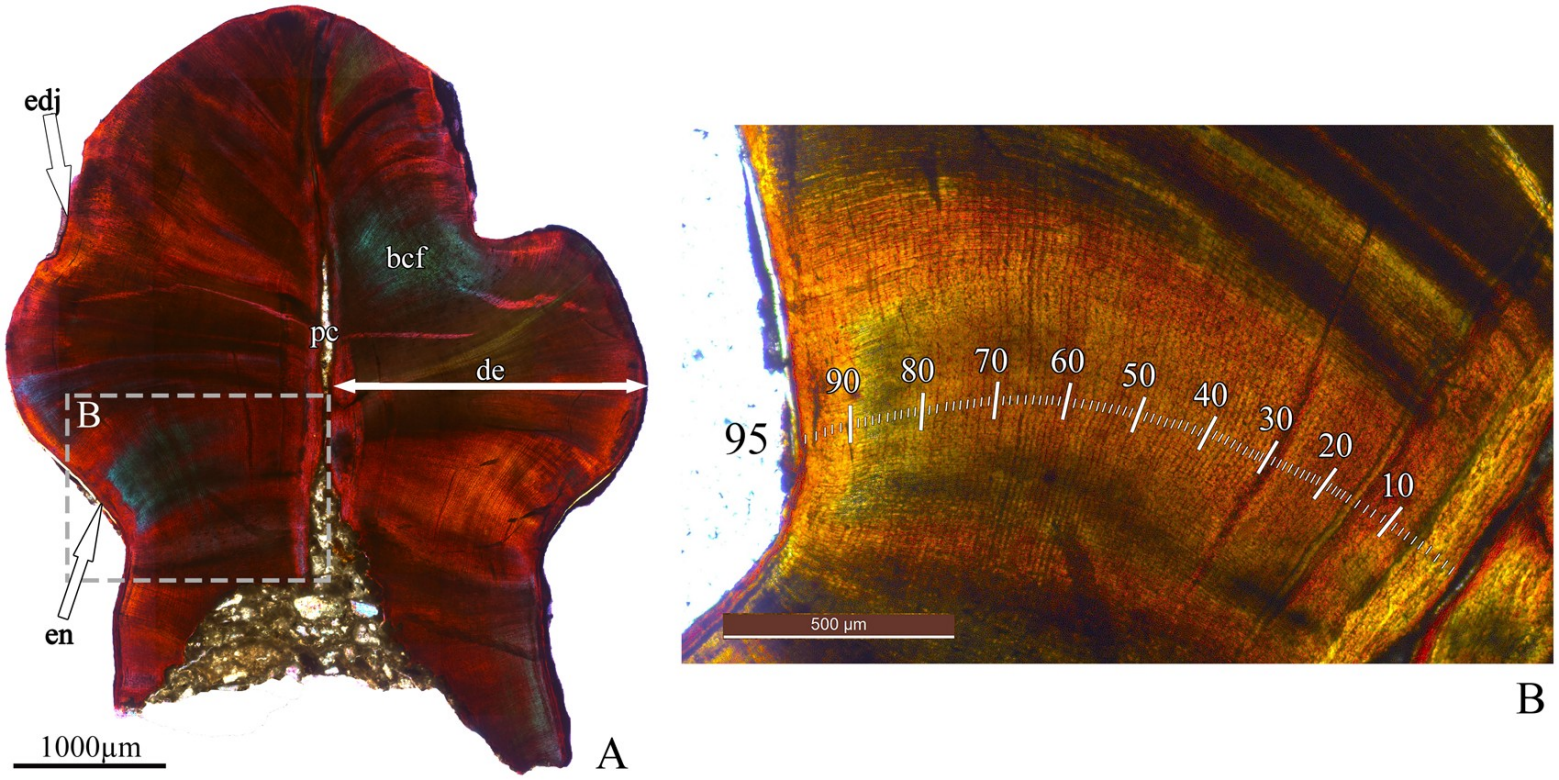
Dental histology of Stegosauria indet. from the Teete locality, Yakutia, Russia; Batylykh Formation (Lower Cretaceous). Thin sections of the specimen ZIN PH 41/246. Coronal section under polarized light with lambda waveplate (A) and close-up of coronal section under normal light (B) showing incremental lines of von Ebner (indicated by white stripes). Abbreviations: en, enamel; de, dentin; cp, pulp cavity; edj, enamel-dentin junction; bcf, bundles of collagen fibres.

On the longitudinal (coronal) and transverse thin sections of ZIN PH 41/246 (Fig 13), the dentin exhibits numerous incremental daily lines (the Lines of von Ebner) correlating with tooth age (see [20]). We counted 95 incremental lines of von Ebner in the coronal section and only 54 in the transverse section of the same tooth ZIN PH 41/246. This discrepancy is in accordance with the observation by D’Emic et al., 2013 [29] who studied tooth replacement rates in sauropods by counting incremental lines and who noticed that in transverse sections the limited number of incremental lines of von Ebner exposed in any given transverse plane.

**Fig 14:**
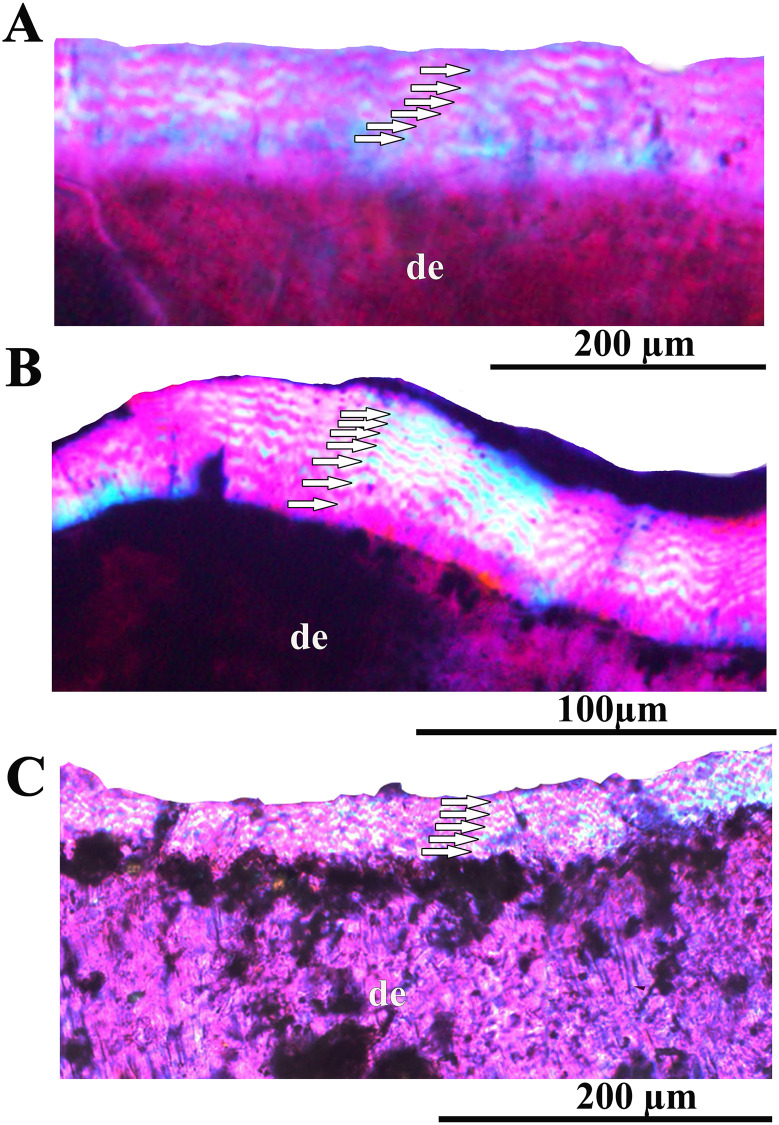
Wavy enamel in stegosaurs and primitive ceratopsians under polarized light with lambda waveplate (“waves” are marked by white arrows). Transverse section of the tooth ZIN PH 38/246 of Stegosauria indet. from the Teete locality, Yakutia, Russia; Batylykh Formation (Lower Cretaceous) (A); transverse section of the tooth ZIN PH 104/117 of Stegosauria indet. from the Berezovsk coal mine, Krasnoyarsk Territory, Western Siberia, Russia (Middle Jurassic) (B); transverse section of the tooth (without number) of *Psittacosaurus* sp. from the Shestakovo locality, Kemerovo Province, Western Siberia, Russia (Lower Cretaceous) (C). Abbreviations: de—dentin.

The enamel of the sectioned tooth ZIN PH 41/246 (Fig 14A) is thin (about 50 μm) and shows a “wavy enamel pattern” which is characteristic for advanced ornithopods ([30] Fig 14A).

## Discussion

### Affinities of the Teete stegosaur

The examined teeth from Teete can be assigned to the Stegosauria based on the following combination of features: the presence of a slightly asymmetrical crown, the presence of a well-developed and pronounced cingulum which is at about the same level on each crown surface, rounded and not sharply pointed tips of the denticles, and denticles that continue onto the adjacent surface of the crown to varying extent, appearing as semicircular, well-marked ridges in cross section (see stegosaurian dental features in [18,23,26]).

The stegosaurian teeth from Teete resemble the teeth of *Stegosaurus* in having a similar crown shape and number of denticles (9–14), in having a pronounced ring-like cingulum, in lacking a well-pronounced primary ridge, and in the presence of a “complex network of secondary ridges” (see descriptions/figures of teeth of *Stegosaurus* in [18,24,25]). Thus, tooth morphology suggests that the Teete stegosaur is likely a derived stegosaur with possible stegosaurine affinities. However, pending the description of the stegosaurian skeletal material from Teete, we conservatively identify the studied stegosaurian teeth as Stegosauria indet.

### Intraspecific and ontogenetic variation

Most of the stegosaurian teeth from Teete closely resemble each other. There are no consistent morphological patterns that would indicate the presence of more than one stegosaurian taxon.

**Fig 15:**
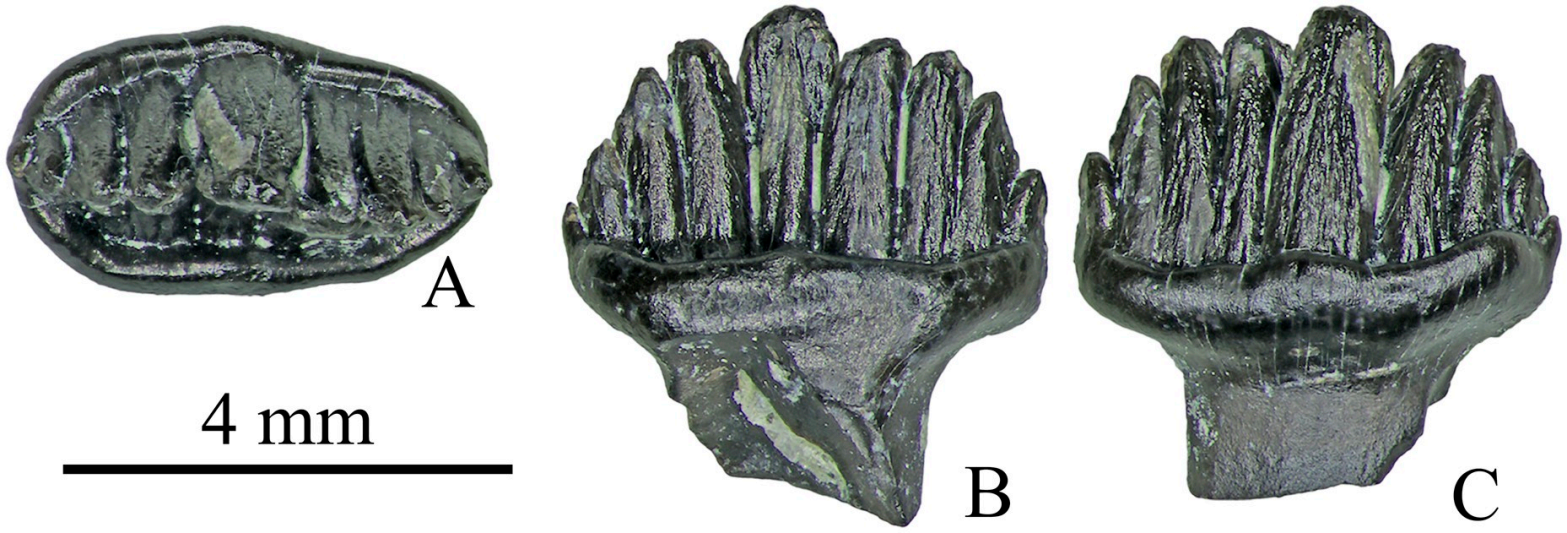
Small dentary tooth ZIN PH 33/246 of Stegosauria indet. from the Teete locality Yakutia, Russia; Batylykh Formation (Lower Cretaceous) in occlusal (A), labial (B) and lingual (C) views.

In the smallest and presumably juvenile dentary tooth (specimen ZIN PH 33/246; mesiodistal length (L) 4.3 mm and labiolingual width (W) 2.4 mm; Fig 15), the main morphological features found in larger teeth such as the presence of a ring-like massive cingulum and the presence of a “complex network of secondary ridges” are present. It differs from the larger specimens in having a lower crown, fewer denticles (nine in ZIN PH 33/246 vs. up to 14 in larger teeth) and in having deeper incisures between the tips of the median and adjacent denticles. We interpret these differences between ZIN PH 33/246 and the larger specimens as ontogenetic variations.

As was reported earlier by Averianov et al., 2019 [10], most of the stegosaur bones collected at Teete belong to juvenile individuals which suggests that stegosaurs permanently lived in this area and reproduced there. The finding of a juvenile stegosaurian tooth ZIN PH 33/246 (Fig 15) at Teete is further support for this suggestion.

### Occlusion and jaw mechanics in the stegosaur from Teete

The tooth wear pattern and dental function in stegosaurs is still poorly known. Gilmore, 1914 [25] noted that many of the tooth crowns of *Stegosaurus* “show marks of wear, being obliquely ground”. Barrett, 2001 [26] noted that some stegosaurian teeth (e.g., some teeth of *Stegosaurus*, one of the dentary teeth of the holotype of *Huayangosaurus*, and several *Paranthodon* maxillary teeth) display wear facets that are likely the result of tooth-food wear. Barrett, 2001 [26] also mentioned that wear facets seen in *Stegosaurus* are (1) small and planar, low angled with respect to the tooth crown, blunt and almost horizontally inclined, or (2) slightly larger with a sharper leading edge and larger exposure of dentin (the same kind of wear facets was also found in *Paranthodon*). The worn dentary tooth of *Huayangosaurus* has a large, almost vertically inclined wear facet on the labial surface of the crown that appears to be the product of tooth-tooth wear [26]. The presence of a large lingual wear facet was reported for *Stegosaurus* cf. *ungulatus* from the Upper Jurassic of Portugal [31]. Later, Billon-Bruyat et al., 2010 [23] described a small wear facet on the apical tip of a tooth of Stegosauria indet. from the Early Cretaceous of France and similar facets for cf. *Stegosaurus armatus* and *Hesperosaurus mjosi* from the Late Jurassic Morrison Formation (USA). In the same year, Reichel, 2010 [32] observed extensive wear facets in some teeth of *Stegosaurus* from the Morrison Formation, suggesting a longer active life of the teeth. Wings et al., 2015 [33] described the presence of an apical wear facet and microwear on a stegosaurian tooth (Stegosauria indet.) from the Middle-Late Jurassic Qigu Formation and concluded that this is morphologically consistent with tooth–tooth occlusion. Later, the presence of wear facets on dentary teeth was reported for *Gigantspinosaurus* from the Late Jurassic of China [34].

Recently, Woodruff et al., 2019 [35] described extensive wear facets on the tooth crowns of a stegosaur from the Morrison Formation (USA) and suggested a complex chewing motion for derived stegosaurs. This conclusion is not consistent with an earlier study of cranial biomechanics by Lautenschlager et al., 2016 [36] who suggested that significant oral processing was unlikely for *Stegosaurus*; studies by Nabavizadeh, 2016 and Nabavizadeh and Weishampel, 2016 [37,38] suggested only very slight palinal motion during chewing in stegosaurs.

Since we only have isolated teeth in the sample, it is difficult to reconstruct the jaw mechanics of the Teete stegosaur in detail. However, the identified tooth macro- and microwear patterns allow to make some suggestions about dental function and jaw action. First of all, the prevalence of worn teeth in the sample and the presence of extensive wear facets of different types (e.g., steeply inclined facets, groove-like facets) suggests that the maxillary and dentary teeth precisely occluded (= tooth-tooth contact). The second important dental feature is the predominance scratches that are oriented mesiodistally or slightly obliquely to the mesiodistal axis. This orientation of scratches, together with mesiodistally oriented groove-like wear facets, suggests the presence of palinal movements of the mandibles. The presence of differently oriented straight and curved scratches on some wear facets also suggests a complex chewing motion.

Accordingly, we suggest a complex jaw mechanism for the Teete stegosaur, where the initial orthal motion was accompanied by a substantial palinal motion (= retractive powerstroke). This is in accordance with hypotheses forwarded by Nabavizadeh, 2016 and Nabavizadeh and Weishampel, 2016 [37,38] on the presence of a palinal motion during chewing in stegosaurs and by Woodruff et al., 2019 [35] on a complex chewing motion for derived stegosaurs.

### Formation of more than one wear facet

The presence of two or even three wear facets on a single tooth crown was not described earlier for Stegosauria (but two facets were figured for a stegosaur from the Morrison Formation (USA), [35]: Fig 10D), and multiple wear facets are possibly unique for the Teete stegosaur. Taking into account the “en échelon” arrangement of maxillary and dentary teeth in stegosaurs, the confluence or overlapping (e.g. groove-like facet within flat apical facet, see Fig 10G and 10H) of wear facets in the Teete stegosaur, and only orthal and palinal movements of the mandibles, we suggest that two or even three wear facets are the result of a consistent contact of the functional tooth with opposite teeth of different ages (with older and with newly erupted replacement tooth/teeth). If our interpretation of the mechanism for the formation of two (three) wear facets is correct, then this observation may be additional evidence for a higher tooth replacement rate in the Teete stegosaur compared to other stegosaurs.

### Tooth formation time

The estimated tooth formation time for ZIN PH 41/246 is 95 days (Fig 13). This is relatively short compared with the tooth formation time known for the majority of other dinosaurs (132–933 days, see [20,29]).

There are at least two possible explanations for the short tooth formation time in the Teete stegosaur (and these explanations do not exclude each other): (1) stegosaurian teeth are relatively small compared to teeth of dinosaurs with longer tooth formation time reported by Erickson, 1996 [20] (ankylosaurs, hadrosaurids, ceratopsids, troodontids, dromaeosaurids, tyrannosaurids,) or by D’Emic et al., 2013 [29] (sauropods), and small teeth need a shorter period of time to be formed; and (2) the Teete stegosaur had a short tooth formation time and, possibly, a high tooth replacement rate, because the teeth were abraded quickly and had to be replaced after short functional life (this suggestion is indirectly supported by the prevalence of worn teeth in the sample). The presence of two (and more) facets on many teeth of the Teete stegosaur also indirectly supports high tooth replacement rates: these teeth were in occlusal contact with opposite teeth of at least two generations during their functional life.

### Wavy enamel pattern

The histological analysis revealed the presence of a “wavy enamel pattern” in the teeth of the Teete stegosaur (Fig 14A). This enamel type was found earlier only in iguanodontians (and it was previously considered as an iguanodontian or dryomorphan synapomorphy) and in the ornithischian dinosaur *Changchunsaurus* [5,30,39,40]. The presence of this enamel type in *Changchunsaurus* showed that this feature is characteristic not only for dinosaurs with complex dentitions, but also for forms with leaf-shaped teeth arranged in one functional row [30]. Chen et al., 2018 [30] also concluded that the wavy enamel type evolved in association with a shearing-type dentition with a roughly symmetrically-enamelled crown, although its precise function is still unclear.

The finding of wavy enamel in the stegosaurian teeth from Teete raised the following questions: (1) is this feature exclusively characteristic for high-latitude stegosaurs (= special adaptation of polar stegosaurs) only or also present in stegosaurs from temperate latitudes? (2) could this feature be characteristic for other forms with leaf-shaped teeth? To answer these questions we studied thin sections of stegosaurian teeth from the Middle Jurassic Itat Formation that formed in temperate latitudes and thin sections of leaf-shaped teeth of the primitive ceratopsian *Psittacosaurus* from the Lower Cretaceous Ilek Formation. We found wavy enamel in the teeth of all mentioned dinosaurs (Fig 14B and 14C). This suggests that wavy enamel is likely characteristic for all stegosaurs (Jurassic and Cretaceous, high-latitude and temperate) and that this feature is common for different ornithischian clades, including ornithopods, marginocephalians and thyreophorans.

### Dental adaptations of high-latitude Teete stegosaurs

The Teete stegosaur was adapted to live in high-latitude conditions and to feed on indigenous plants, but it is not clear which of the described dental features are unique for this species and represent adaptations for “polar life”, and which are common within the stegosaurian clade. According to the palynological analysis by Kolosov et al., 2009 [16], the flora of Teete includes mosses, lycophytes, ferns and different conifers (Pinaceae indet., *Pinus* sp., *Picea* sp., *Podocarpus multesima*), and these plants (including coarse conifers) probably were part of the Teete stegosaur diet. The high degree of tooth wear in the Teete stegosaur may have been partly caused by abrasive components (e.g., resistant stems and woody branches) in the diet.

Previous studies of cranial biomechanics of stegosaurs have shown that *Stegosaurus* (low-latitude form) possessed relatively high bite forces (231–410 N or 166–321 N according to different models) and only moderate stress magnitudes, which indicates that they would have been capable of foraging on a wide variety of different vegetation types and plant matter ([36]; see also [32] for previous cranial biomechanics study of *Stegosaurus*). The study of stegosaurs from the low-latitude Morrison Formation revealed a high degree of dental wear and possible complex chewing motions in derived stegosaurs [35].

Up to date, the Teete stegosaurian teeth exhibit the highest degree of wear compared to other stegosaurian taxa and this, along with the presence of up to three wear facets, short time of tooth formation and possibly high tooth replacement rate, potentially indicates a specific adaptation to a diet of coarse plant materials in this high-latitude form. However, the degree of tooth wear and the complexity of jaw movements are unknown in most stegosaurs and some taxa, including derived stegosaurines (see [35], “Occlusion and jaw mechanism in stegosaur from Teete” above), could have had a similar dental adaptation. In this case the features of the Teete taxon such as the high degree of tooth wear, more than one wear facet on a single tooth, complex jaw motions (both features also known in a stegosaur from the low-latitude Morrison Formation [35]), short tooth formation time and possibly high tooth replacement rate, together with relatively high bite forces found in *Stegosaurus* [36], are possibly common stegosaurian adaptations which allowed stegosaurs to be a successful group of herbivorous dinosaurs in Middle Jurassic–Early Cretaceous time and to live in both low- and high-latitude ecosystems.

## Conclusions

The study of isolated stegosaurian teeth from the Early Cretaceous Teete locality in Yakutia (Eastern Siberia, Russia) revealed several dental features, which were not observed in stegosaurs before or were reported only for a limited number of taxa. These features are: (1) high degree of dental wear (presence of small apical and/or steeply inclined facets was reported for many stegosaurian teeth but the prevalence of strongly worn teeth was not found among stegosaurs earlier); (2) presence of several types of facets, which indicates tooth-tooth contact and precise dental occlusion; (3) short tooth formation time; and (4) presence of “wavy enamel pattern” (stegosaurian teeth were not examined histologically before). Additionally, the complex microwear pattern suggesting complex, multiphase jaw movements with a palinal jaw motion reported here for the Teete stegosaur/stegosaurs, was found earlier only in a stegosaur from the Morrison Formation (USA).

As a result, the suite of dental features found in the Teete stegosaurian teeth could be (1) a unique adaptation for a life in “harsh” high-latitude subpolar conditions with limited available variety of plants and predominance of coarse and resistant plant materials (e.g., branches of conifers) in the diet, or (2) a combination of characters typical for stegosaurs that remain largely unobserved. For testing these hypotheses, studies of stegosaur tooth morphology and histology, macro- and microwear, variations along the tooth row, jaw movements and tooth replacement rates are strongly needed.

## Supporting information

S1 File Measurements of studied maxillary teeth. (XLS)

S2 File Measurements of studied dentary teeth. (XLS)

## References

[1] Buffetaut E. Polar dinosaurs and the question of dinosaur extinction: a brief review. Palaeogeogr Palaeoclimatol Palaeoecol 2004;214: 225–231.

[2] Fiorillo AR, Gangloff RA. Theropod teeth from the Prince Creek Formation (Cretaceous) of Northern Alaska, with speculations on arctic dinosaur paleoecology. J Vertebr Paleontol. 2009;20: 675–682.

[3] Fiorillo AR, Gangloff RA. The caribou migration model for Arctic hadrosaurs (Dinosauria: Ornithischia): a reassessment. Hist Biol. 2001;15: 323–334.

[4] Fiorillo AR, McCarthy PJ, Flaig PB. Taphonomic and sedimentologic interpretations of the dinosaur-bearing Upper Cretaceous strata of the Prince Creek Formation, Northern Alaska: insights from an ancient high-latitude terrestrial ecosystem. Palaeogeogr Palaeocl Palaeoecol. 2010;295: 376–388.

[5] Godefroit P, Golovneva L, Shchepetov S, Garcia G, Alekseev P. The last polar dinosaurs: High diversity of latest Cretaceous Arctic dinosaurs in Russia. Naturwissenschaften. 2009;96(4): 495–501. 10.1007/s00114-008-0499-0 19089398

[6] Poropat SF, Martin SK, Tosolini A-MP, Wagstaff BE, Bean LB, Kear BP et al. Early Cretaceous polar biotas of Victoria, southeastern Australia—an overview of research to date. Alcheringa. 2018. 10.1080/03115518.2018.1453085

[7] Rich THV, Gangloff RA, Hammer WR. Polar dinosaurs. In: Currie PJ, Padian K, editors. Encyclopedia of Dinosaurs. San Diego, CA: Academic Press; 1997. pp. 562–573.

[8] Rich THV, Vickers-Rich P, Gangloff RA. Polar dinosaurs. Science. 2002; 295: 979–980. 10.1126/science.1068920 11834803

[9] Averianov AO, Martin T, Lopatin AV, Skutschas PP, Schellhorn R, Kolosov PN et al. A highlatitude fauna of mid-Mesozoic mammals from Yakutia, Russia. PLoS ONE 2018;13: e0199983. 10.1371/journal.pone.0199983 30044817 PMC6059412

[10] Averianov AO, Skutschas PP, Schellhorn R, Lopatin AV, Kolosov PN, Kolchanov VV et al. The northernmost sauropod record in the Northern Hemisphere. Lethaia. 2019. 10.1111/let.12362

[11] Averianov AO, Martin T, Lopatin AV, Skutschas PP, Schellhorn R., Kolosov P et al. A new euharamiyidan mammaliaform from the Lower Cretaceous of Yakutia, Russia. J Vertebr Paleontology. 2019;39(6): e1762089.

[12] Lopatin AV, Agadjanian AK. A tritylodont (Tritylodontidae, Synapsida) from the Mesozoic of Yakutia. Dokl Biol Sci. 2008;419(1): 107. 10.1134/s0012496608020117 18536275

[13] Skutschas PP, Kolchanov VV, Averianov AO, Martin T., Schellhorn R, Kolosov PN et al. A new relict stem salamander from the Early Cretaceous of Yakutia, Siberian Russia. Acta Paleontol Pol. 2018;63(3): 519–525.

[14] Skutschas PP, Markova VD, Kolchanov VV, Averianov AO, Martin T, Schellhorn R.et al. Basal turtle material from the Lower Cretaceous of Yakutia (Russia) filling the gap in the Asian record. Cretaceous Res. 2020;106: e104186. 10.1016/j.cretres.2019.07.016

[15] Kurzanov SM, Efimov MB, Gubin YM. New archosaurs from the Jurassic of Siberia and Mongolia. Paleontol J. 2003;37: 53–57.

[16] Kolosov PN, Ivensen GV, Mikhailova TE, Kurzanov SM, Efimov MB, Gubin YM. Taphonomy of the Upper Mesozoic tetrapod Teete locality (Yakutia). Paleontol J. 2009. 10.1134/S0031030109020129

[17] Maidment SCR, Norman DB, Barrett PM, Upchurch P. Systematics and phylogeny of Stegosauria (Dinosauria: Ornithischia). J Syst Palaeontol. 2008;6: 364–407.

[18] Galton PM, Upchurch P. Stegosauria. In: Dodson P, Osmólska H, Weishampel DB, editors. The Dinosauria. Berkeley: University of California Press, 2004. pp 343–362.

[19] Tumanova TA, Alifanov VR. First record of stegosaur (Ornithischia, Dinosauria) from the Aptian–Albian of Mongolia. Paleontol J 2018; 52: 1771–1779.

[20] Erickson GM. Incremental lines of von Ebner in dinosaurs and the assessment of tooth replacement rates using growth line counts. Proc Natl Acad Sci USA. 1996;93: 14623–14627. 10.1073/pnas.93.25.14623 8962103 PMC26184

[21] Averianov AO, Martin T, Skutschas PP, Danilov IG, Schultz J, Schellhorn R et al. Middle Jurassic vertebrate assemblage of Berezovsk coal mine in Western Siberia (Russia). Global Geology. 2016;19(4): 187–204.

[22] Averianov AO, Voronkevich AV, Leshchinskiy SV, Fayngertz AV. A ceratopsian dinosaur *Psittacosaurus sibiricus* from the Early Cretaceous of West Siberia, Russia and its phylogenetic relationships. J Syst Palaeontol. 2006;4(4): 359–395.

[23] Billon-Bruyat J-P, Mazin J-M, Pouech J. A stegosaur tooth (Dinosauria, Ornithischia) from the Early Cretaceous of southwestern France. Swiss J Geos. 2010;103(2): 143–153.

[24] Galton PM. Teeth of ornithischian dinosaurs (mostly Ornithopoda) from the Morrison Formation (Upper Jurassic) of the western United States. In: Carpenter K, editor. Horns and Beaks: Ceratopsian and Ornithopod Dinosaurs. Bloomington&Indianapolis: Indiana University Press, 2007. pp. 17–47.

[25] Gilmore CW. Osteology of the armored Dinosauria in the Unites States National Museum, with special reference to the genus *Stegosaurus*. Bull U.S. Natl Mus. 1914;89: 1–143.

[26] Barrett PM. Tooth wear and possible jaw action of *Scelidosaurus harrisonii* Owen and a review of feeding mechanisms in other thyreophoran dinosaurs. In: Carpenter K, editor. The Armored Dinosaurs. Bloomington: Indiana University Press, 2001. pp. 25–52.

[27] Ősi A, Barrett PM, Fӧldes T, Tokai R. Wear pattern, dental function, and jaw mechanism in the late Cretaceous Ankylosaur *Hungarosaurus*. Anatom Rec. 2014;297: 1165–1180. 10.1002/ar.22910 24700688

[28] Averianov AO, Krasnolutskii SA. Stegosaur remains from the Middle Jurassic of West Siberia. Proc Zool Inst Russ Acad Sci. 2009;313: 153–167.

[29] D’Emic MD, Whitlock JA, Smith KM, Fisher DC, Wilson JA. Evolution of High Tooth Replacement Rates in Sauropod Dinosaurs. PLoS ONE. 2013; 8(7): e69235. 10.1371/journal.pone.0069235 23874921 PMC3714237

[30] Chen J, LeBlanc ARH, Jin L, Huang T, Reisz RR. Tooth development, histology, and enamel microstructure in *Changchunsaurus parvus*: Implications for dental evolution in ornithopod dinosaurs. PLoS ONE. 2018;13(11): e0205206. 10.1371/journal.pone.0205206 30403689 PMC6221265

[31] Escaso F, Ortega F, Dantas P, Malafaia E, Pimentel NL, Pereda-Suberbiola X, et al. New evidence of shared dinosaur across Upper Jurassic Proto-North Atlantic: *Stegosaurus* from Portugal. Naturwissenschaften 2007;94: 367–374. 10.1007/s00114-006-0209-8 . 17187254

[32] Reichel M. A model for the bite mechanics in the herbivorous dinosaur Stegosaurus (Ornithischia, Stegosauridae). Swiss J Geos. 2010;103: 235–240.

[33] Wings O, Tütken T, Fowler DW, Martin T, Pfretzschner H, Sun G. Dinosaur teeth from the Jurassic Qigu and Shishugou Formations of the Junggar Basin (Xinjiang/China) and their paleoecologic implications. Palӓontol Z. 2015;89: 485–502. 10.1007/s12542-014-0227-3

[34] Hao B, Zhang Q, Peng G, Ye Y, You H. Redescription of *Gigantspinosaurus sichuanensis* (Dinosauria, Stegosauria) from the Late Jurassic of Sichuan, Southwestern China. Acta Geol Sin-Eng. 2018;92: 431–441.

[35] Woodruff DC, Trexler D, Maidment SCR. Two new stegosaur specimens from the Upper Jurassic Morrison Formation of Montana, USA. Acta Palaeontol Pol. 2019;64(3): 461–480.

[36] Lautenschlager S, Brassey CA, Button DJ, Barrett PM. Decoupled form and function in disparate herbivorous dinosaur clades. Sci Rep-UK. 2016;6: 26495. 10.1038/srep26495 27199098 PMC4873811

[37] Nabavizadeh A. Evolutionary trends in the jaw adductor mechanics of ornithischian dinosaurs. Anat Rec. 2016;299: 271–294. 10.1002/ar.23306 26692539

[38] Nabavizadeh A, Weishampel DB. The Predentary Bone and Its Significance in the Evolution of Feeding Mechanisms in Ornithischian Dinosaurs. Anat Rec. 2016;299: 1358–1388. 10.1002/ar.23455 27490958

[39] Becerra MG, Pol D. The enamel microstructure of *Manidens condorensis*: New hypotheses on the ancestral state and evolution of enamel in Ornithischia. Acta Palaeontol Pol. 2020;65(X): xxx–xxx.

[40] Hwang SH. The evolution of dinosaur tooth enamel microstructure. Biol Rev Camb Philos. 2011;86(1): 183–216. 10.1111/j.1469-185X.2010.00142.x 20518758

